# The Relationship Among Spirituality, Fear, and Mental Health on COVID-19 Among Adults: An Exploratory Research

**DOI:** 10.3389/fpsyg.2021.815332

**Published:** 2022-01-12

**Authors:** Balan Rathakrishnan, Soon Singh Bikar Singh, Azizi Yahaya, Mohammad Rahim Kamaluddin, Siti Fardaniah Abdul Aziz

**Affiliations:** ^1^Faculty of Psychology and Education, University Malaysia Sabah (UMS), Kota Kinabalu, Malaysia; ^2^Centre for Research in Psychology and Human Well-Being, Faculty of Social Sciences and Humanities, National University of Malaysia, Bangi, Malaysia

**Keywords:** spirituality, fear, mental health, COVID-19, adults

## Abstract

The novel coronavirus disease (COVID-19) is impactful on all aspects of individuals’ lives, particularly mental health due to the fear and spirituality associated with the pandemic. Thus, purpose of this study was to identify the relationship among fear, spirituality, and mental health on COVID-19 among adults in Malaysia. This study also examines spirituality as a mediator in relationship between fear and mental health. The study involved around 280 adults in Malaysia. This research is a quantitative study. Data analysis method (SEM-PLS) has been used for data analysis. Based on descriptive analysis, mental health questionnaire indicated that 60.0% of them are at a poor level of mental health whereas 57.5% of respondents showed a moderate level of COVID-19 fear, and 60.4% of respondents owned moderate level of spiritual well-being. The results also demonstrated that respondents that have a high level of fear would have a high level of mental health; interestingly, those with a high level of spirituality will have a lower level of mental health. Findings indicated that spirituality significantly mediated the relationship between fear and mental health. This research will help to demonstrate how important spirituality values to control mental health to be more positive among adults in Malaysia. The main contributions of this study are to help come out with new intervention method for those who are mentally ill and need help.

## Introduction

In early 2020, coronavirus disease (COVID-19), originating from Wuhan in Hubei province, began to spread throughout China (Li et al., 2020). World Health Organization (WHO) stated that there is a high risk of COVID-19 spreading to other countries worldwide. In March 2020, WHO assessed that COVID-19 could be characterized as a pandemic (WHO, 2020). As a result, the majority of countries in the world have to take drastic measures to adopt a nationwide lockdown and practice social distancing to fight against and to flatten the curve of the COVID-19 infection. The spread of the disease without any vaccine and uncertainty and low expectations of COVID-19 not only threaten people’s physical health but also poses challenges to people’s mental health for the entire human race. For instance, in Italy, local people have high levels of stress due to no firm estimate of how long the pandemic will last and how long our lives will be disrupted or whether or not our loved ones or we will be infected (Montemurro, 2020).

In China, fear of abandonment, neglected death toll increase among patients, and feelings of loneliness and anger have developed among people who are quarantined (Xiang et al., 2020). Research by Zhang et al., 2020 shows that being quarantined decreases face-to-face connection and social interaction, and it contributes to the stressful situation among people. Besides, in India, most of the people are in fear, disgust, stress, and extreme sadness about the lockdown on March 25, 2020 (Barkur and Vibha, 2020), sleep difficulties, and paranoia about acquiring COVID-19 infection (Roy et al., 2020). One of the reasons is social isolation which can cause people to get stress (Holt-Lunstad et al., 2015).

Even though many coping techniques and procedures have been formulated for COVID-19, past studies have proved that spirituality values improve people’s well-being. Richards and Bergin (2005) and Young et al. (2007) have demonstrated the connection between spirituality with mental health and well-being. Spirituality has helped people with making decisions and helping people to cope with stress when they have difficulty in life (Pargament et al., 1988; Thurston, 1999). When people are practicing spirituality values, they can overcome depression (Westgate, 1996; Srinivasan et al., 2003) and anxiety (Graham et al., 2001). Brown et al. (2013) in his research have proven that spirituality well-being has a significant effect on anxiety and depression and those who have a higher level of spirituality have a reduction in their mental illness. Furthermore, some research studies have been performed to investigate how spirituality could help people to improve their self-esteem, giving them moral support and searching meanings of life among patients with cancer (Thune-Boyle et al., 2006).

Another critical element that needs to be looked into is the fear of illness. Fear is related to the contemporary mental health system (Laurance, 2003). Fear is defined as the unpleasant emotional state that is elicited by a perceived threat (de Hoog et al., 2008). Due to the lack of control on the pandemic in terms of unavailability of an effective vaccine and treatment cure, individuals naturally began experiencing fear regarding developing the disease. Literature suggests that whereas fear of COVID-19 propels individuals to observe the rules that will help to minimizing the spread of the virus, it may also result in an array of psychological effect such as anxiety (Roy et al., 2020) and depression (Holmes et al., 2020). Fear is also linked with mental illness with different types of sociocultural factors such as our belief and dominant culture (Halliwell, 2009). Previous studies have highlighted that there is a significant relationship between the fear of illness and mental health (Laurance, 2003). Even though fear and mentality are positively related, but there is very few research that explores the relationship among fear of COVID-19, the value of spirituality, and mental health among adults in Malaysia. These three aspects are closely related, but how can they be seen during the COVID-19 pandemic. Even though spirituality and fear have been discussed in the past, there is still the question of how spirituality could help people who have mental illness and fear of disease. Research is still lacking regarding the pandemic, notably the COVID-19 pandemic.

The main aim of this study was to examine the relationship between fear of COVID-19 and mental health with an adult sample as mediated by spirituality. The hypotheses of the study included that (a) there is significant relationship between fear and spirituality, (b) there is significant relationship between spirituality and mental health, (c) there is significant relationship between fear and mental health, and (d) spirituality mediates the relationship between fear and mental health. As no studies per see examined such link between the concepts, exploring such relationship is certainly worth studying.

## Literature Review

### Relationship Among Fear, Spirituality, and Mental Health

According to behavioral explanations, spirituality and health are much related because of people behaviors, motivation, belief, attitudes, and thoughts. Physical and mental health are related to psychosocial variables, such as stress, lifestyle behaviors, and health-related cognitions. This helps us to understand how the practice of faith and spiritual path may import impact on psychological health. Individual prayers or worship may produce solution for emotions such gratitude, humility, forgiveness, and some of them be fear of doing bad things. As conclusion, the connections among mental health, spirituality, and fear are described (Levin, 2009).

### Relationship Between Fear and Spirituality

Previous research has proved that those who have a fear of diseases but have spirituality values have positive mental health (Hayman et al., 2007; Koenig, 2010). Study by Fardin (2020) indicated that spiritualty aids people to have mental relaxation in times of crisis. Some of the religious solutions proposed against the COVID-19 prevalence could be helpful. This shows that spirituality values can help people who have a fear of coping with difficult situations. Thus, this research proposes the hypothesis below:

H1: There is significant relationship between fear and Spirituality.

### Relationship Between Spirituality and Mental Health

Spirituality is unique in its definition because there is no precise definition of spirituality. Spirituality is looking for meaning in life, peace, bliss, understanding, faith, and love. Rosmiran (2020) mentioned that she has been talking and advising her patients to always have faith and hope to overcome their mental issue, especially during the COVID-19 pandemic. Even patients have been anxious about their life in this pandemic but hope and faith could help them to overcome their mental health state more positively. This proves how much spirituality values could boost up more positive mental health. Many past researches have discussed the relationship between spirituality and mental health (Seybold and Hill, 2001; Miller and Thoresen, 2003; Powell et al., 2003). Thus, this research proposes the hypothesis below:

H2: There is significant relationship between spirituality and mental health.

### Relationship Between Fear and Mental Health

The fear of COVID-19 is undoubtedly affecting mental health condition of people. Research by Zhang et al., 2020 shows that during quarantine, people lose face-to-face connection and social interaction, and it contributes to a stressful situation among people. Fear can cause many mental health issues because people get worries related to disease and also because they have been isolated at home for a more extended period. People who already have some mental health issues will be more exceptional in having mental health risks such as fear and anxiety. This statement has been proven by Druss (2020). Fear is not only related to disease but also toward social distancing, and this has been proven by Holt-Lunstad et al. (2015), whereby social distancing can cause many health problems such as fear, guilt, unhappiness, and depression. His finding shows how much fear can contribute to mental health, and it becomes worse for those who already have some mental health issues. In China, fear of abandonment, being neglected and rising death among patients, and feelings of loneliness and anger have developed among people who are quarantined (Xiang et al., 2020). When social distancing was practiced in Malaysia, many adults feel that they are alone and are influenced by the many social media information. Thus, this research proposes the hypothesis below:

H3: There is significant relationship between fear and mental health.

### Spirituality as a Mediator

Based on previous study, spirituality is the important variable as a mediator (Reutter and Bigatti, 2014; Trigwell et al., 2014). Spirituality values can help people to cope with fear and mental health issue. When spirituality values are high, people give less importance to emotion and become more focused on daily work. Even if people are experiencing social distancing and also having less social connectedness with their friends, family members, and colleagues, with spiritually, they can have faith and hope. This spirituality reduces their fear of disease. Spirituality makes them stronger in their relationship with fear and mental health (Bonelli et al., 2012). Furthermore, in Malaysia, adults are multiethnic whereby each of them has different faith and belief systems in society (Rathakrishnan et al., 2021). In light of these results, it is possible to conclude that spirituality assists individuals to cope better with life disruptions and allows them to view life more positively. Thus, this research proposes the hypothesis below:

H4: Spirituality mediates the relationship between fear and mental health.

## The Research Framework

**FIGURE 1:**
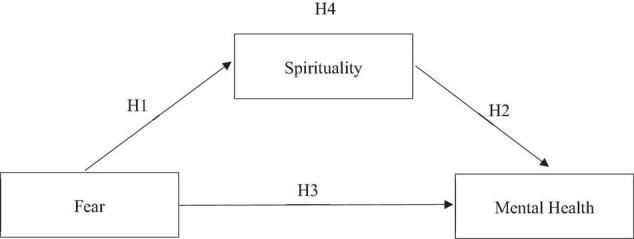
Research framework. Obtained from Universiti Malaysia Sabah.

Figure 1 indicates the research constructs and the operationalization of the constructs based on the past studies.

Figure 1 shows the research framework of the study on how the fear impacts on spirituality with mental health, and spirituality becomes a mediator in relationship between fear and mental health. This is very crucial which concerns the spirituality of awareness and understanding of what issue the adults experiencing in their mental state and how it is related to fear also become an important element to consider necessary during the COVID-19 pandemic. Stemmed from the above framework, this research, therefore, derived a few hypotheses that are constructed as follows:

H1: There is significant relationship between fear and spirituality.
H2: There is significant relationship between spirituality and mental health.
H3: There is significant relationship between fear and mental health.
H4: Spirituality mediates the relationship between fear and mental health.

## Research Methodology

### Research Design

This study has used convenient sampling design, and the total of 280 questionnaires was received from the respondents. Random sampling is the purest form of probability sampling. Each member of the population has an equal chance of being selected. This simple random sampling design has the least bias and offers the most generalizability (Sekaran and Bougie, 2013).

### Sample

In this study, the population is the adults in Malaysia. Adults are classified as individuals who are above the age of 18 years. In 2019, there were approximately 21.82 million adults in Malaysia. According to Raosoft calculator the with the 90% and 0.05 is 271 sample size.

### Instruments and Measures

Spiritual well-being questionnaire (SWBQ) was a set of self-rating questionnaires designed to measure personal’s well-being, communal well-being, environmental well-being, and transcendental well-being (Gomez and Fisher, 2003). It consisted of a total of 20 items, containing five items for each domain mentioned previously. It used a four-point Likert scale (1 = very low, 2 = low, 3 = high, and 4 = very high).

Fear of COVID-19 Scale (FCV-19S) was seven items scale for measuring fear of COVID-19 (Ahorsu et al., 2020). The response type was modified from a five-point Likert scale to a four-point Likert scale (1 = strongly disagree, 2 = disagree, 3 = agree, and 4 = strongly agree).

Patient Health Questionnaire for Depression and Anxiety (PHQ-4) was used to measure respondents’ mental health in terms of depression and anxiety (Kroenke et al., 2009). It consists of four items where two items are taken from Generalized Anxiety Disorder-7 (GAD-7) and another two from the Patient Health Questionnaire-8 (PHQ-8). It uses a five-point Likert scale (0 = not often at all, 1 = not so often, 2 = somewhat often, 3 = very often, and 4 = extremely often). The greater the score in PHQ-4, the greater the level of depression and anxiety, and hence the lower the level of mental health. For this research, all measures for SWBQ, FCV-19, and PHQ-4 have been used 3 scales (low, moderate, and high) as suggested by Mohd Majid (2002).

#### Procedure

In this study, the respondents were recruited using a convenience sampling technique. The entire study was obtained ethical approval board reference to JKEtika 3/20 (11) from the Ethical Board of University Malaysia Sabah. Prior to data collection, signed consent was obtained from each respondent, and they were assured with anonymity and confidentially of data. A total of 280 respondents were selected for the purpose of this research.

#### Data Analysis

Data analysis method (SEM-PLS) has been used for data analysis.

## Results and Discussion

Table 1 shows that about one-third (36.1%) of the respondents were men and the other 63.9% were women. In this survey, 198 (70.7%) of the respondents are aged from 18 to 35 years old and 82 (29.3%) aged from 36 to 56 years old with the mean age of 29.5. Most respondents (68.2%) were single, 31.4% married, and 0.4% divorced. Totally, 171 (61.1%) of the respondents were undergraduate, 66 (23.6%) were graduate, and 43 (15.4%) were postgraduate. In addition, 204 (72.9%) respondents stayed in urban areas, whereas 76 (27.1%) in rural areas.

**TABLE 1:** Distribution of respondents.

	Frequency	Percentage (%)
**Gender**		
Male	101	36.1
Female	179	63.9
**Age group**		
18–35 years	198	70.7
36–56 years	82	29.3
**Marital status**		
Single	191	68.2
Married	88	31.4
Divorced	1	0.4
**Education**		
Undergraduate	171	61.1
Graduate	66	23.6
Postgraduate	43	15.4
**Location**		
Urban	204	72.9
Rural	76	27.1

Based on descriptive analysis as shown in Table 2, most respondents (41.4%) self-reported a good level of mental health, whereas the mental health questionnaire indicated that 60.0% of them are at a good level of mental health. Overall, 96.4% of respondents showed a moderate level of COVID-19 fear, and 60.4% of respondents owned a moderate level of spiritual well-being. About half of the respondents (50.7%) indicated a moderate level of spiritual transcendence.

**TABLE 2:** Descriptive analysis on mental health, fear of COVID-19, and spirituality well-being (*N* = 280).

Variables	Frequency	Percentage (%)
**Self-report mental health status**		
Poor	9	3.2
Fair	51	18.2
Good	116	41.4
Very Good	71	25.4
Excellent	33	11.8
**PHQ-4**		
Good	168	60.0
Moderate	99	35.4
Poor	13	4.6
**FCV-19S**		
Low	4	1.4
Moderate	270	96.4
High	6	2.1
**SWBQ**		
Low	11	3.9
Moderate	169	60.4
High	100	35.7

### Data Analysis Approaches

In this study herein, the structural equation model used partial least squares regression (PLS regression) path model to verify research hypotheses regarding the effect of spirituality between the fear of COVID-19 and mental health. For analysis tool, the SmartPLS 2.0 program was used. The PLS regression path model stands for a structural equation model. It is based on principal component, that is, total dispersion. This technique can evaluate measurement for validity of variable and structural model (Hair et al., 2016).

#### Measurement Model Analysis

In this study, to analyze measurement models, the confirmatory factor analysis (CFA) was conducted. The results are derived from analysis on the PLS measurement model. The convergent validity, internal consistency, and discriminant validity were analyzed the measurement model to evaluate and identify the suitability of them (Hair et al., 2016).

##### Convergent Validity

Convergent validity can be comprehended through individual measuring items for reliability purpose. For individual measuring items with reliability, the loading values should be 0.7 ideally, and 0.6 at minimum in Table 3 (Hair et al., 2016).

**TABLE 3:** Result of crossloading/loadings.

	Fear	Mental health	Environmental	Transcendental	Personal	Communal
F1	0.720					
F3	0.789					
F7	0.758					
MH1		0.807				
MH2		0.806				
MH3		0.821				
MH4		0.777				
S11			0.942			
S13			0.948			
S15			0.919			
S17				0.832		
S18				0.852		
S19				0.828		
S2					0.886	
S5					0.754	
S6						0.880
S10						0.777

As shown in Table 1, all the individual measuring items’ value is above 0.720, in which all the items exceed 0.70 or above. Thus, all the measuring items used in this study are valid and indicate that all items secured convergent validity.

##### Internal Consistency

As internal consistency is a level of validity in which a latent variable set of specific observed variable reflects latent variable, Cronbach’s alpha, average variance extracted (AVE), and composite reliability were used to analyze the internal consistency of measuring model. Generally, it has reliability if it is 0.6 or above in Cronbach’s alpha and if it is 0.5 or above in AVE value, and it has internal consistency if it is 0.7 or above in composite reliability. As the internal consistency shown in Table 4, all the items exceed the above-stated threshold, which secures internal consistency (Hair et al., 2017).

**TABLE 4:** Internal consistency.

Constructs	Composite reliability	Cronbach alpha	Average variance extracted (AVE)
Communal	0.815	0.600	0.689
Environmental	0.955	0.930	0.877
Fear	0.800	0.626	0.572
Mental Health	0.879	0.819	0.645
Personal	0.807	0.600	0.677
Transcendental	0.875	0.787	0.701

##### Discriminant Validity

The level of discriminating a concept of a specific latent variable from a concept of other latent variables is called discriminant validity. In this study, a variable has validity if it uses a square root value of mean dispersion extracted value of all the extracted variables, and an AVE square root value is higher compared with correlation coefficient. The AVE value should 0.70 or above (Hair et al., 2017).

The establishment of discriminant validity of the constructs was presented in Table 5. The threshold criteria at below 1 have achieved to all constructs (Henseler et al., 2016). Thus, it shows that each construct achieved the discriminant validity.

**TABLE 5:** Discriminant validity.

	Communal	Environmental	Fear	Mental health	Personal	Transcendental
Communal	0.830					
Environmental	0.774	0.936				
Fear	–0.658	–0.575	0.756			
Mental Health	–0.257	–0.200	0.297	0.803		
Personal	0.762	0.797	–0.744	–0.319	0.823	
Transcendental	0.469	0.448	–0.605	–0.327	0.542	0.837

#### Structural Model Analysis

The structural model evaluates variance explanation power (*R*^2^) of structural concept and also evaluates significance of path coefficient (β) expressing causal relationship information between two variables through structural equation analysis.

In Table 6, the finding that presented H1 strongly supports (standardized beta = −0.761, *p* = 0.00) that the fear has significant effects on spirituality, and H2 was supported the spirituality significant effect on mental health (standardized beta = −0.212, *p* = 0.03).

**TABLE 6:** Result bootstrapping.

Hypothesis	Path	Beta	S.D	*t*-value	*p*-values	Results	*f* ^2^	*R* ^2^
H1	FR → SPY	–0.761	0.033	23.070	0.00	Supported	1.373	0.579
H2	SPY → MH	–0.212	0.099	2.139	0.03	Supported	0.021	0.107
H3	FR → MH	0.136	0.103	1.325	0.18	Not Supported	0.002	

**FIGURE 2:**
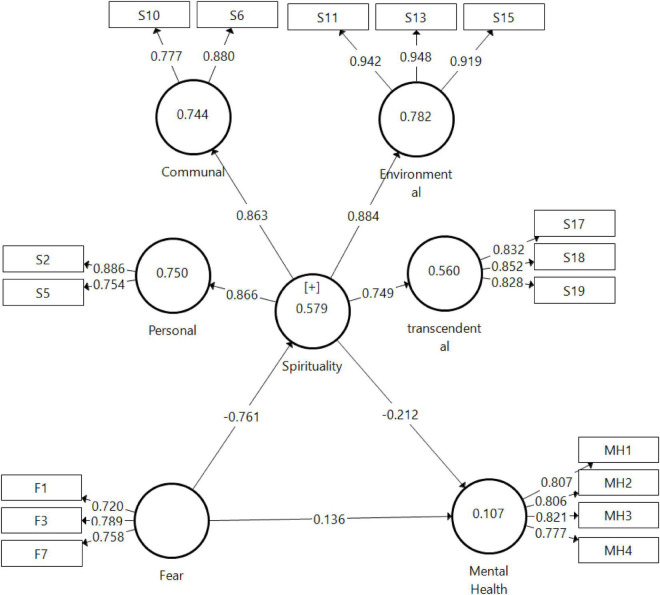
Structural model analysis results. Obtained from Universiti Malaysia Sabah.

Meanwhile, in H3, there was no significant association found between fear and mental health (standardized beta = 0.136, *p* = 0.18). The findings also show that the modeled constructs explain substantial variances in endogenous constructs with good predictive relevance. R2 values were found to be at substantial level for one endogenous construct: SPY (*R*^2^ = 57.9%); and two endogenous constructs: MH (*R*^2^ = 10.7%). Figure 2 demonstrates the structural and measurement models of this study.

### Mediation Effect of Spirituality Between Fear and Mental Health

Table 7 presents the results of hypothesis testing for the indirect path. The findings in Table 7 concluded a significant indirect effect of spirituality on the relationship between fear and mental health (β = 0.161, *t*-value = 2.156, *p*-value = 0.03). The results confirmed that spirituality is a mediator that completely mediate the effects of fear on mental health, and thus it supports H4.

**TABLE 7:** Mediation effect result.

Path	Hypothesis	Indirect Effects				Results
		Beta	Standard deviation	*t*-value	*p*-value	
FR → SPY mediated by MH	H4	0.161	0.075	2.156	0.03	Supported

***p < 0.001; SPY, spirituality; FR, fear; MH, mental health.

Coronavirus disease pandemic is impactful on people’s life due to several reasons such as lockdowns (Meda et al., 2020) and associated isolation (Hwang et al., 2020), fear of worthlessness, and fear of infection (Dubey et al., 2020). Two of the critical antecedents of such mental health problems might include fear of COVID-19 and spirituality. Thus, this study aimed to investigate whether (a) fear of COVID-19 is related to spirituality, (b) spirituality is related to mental health, (c) fear of COIVD-10 is related to mental health, and (d) whether spirituality mediates the relationship between fear of COVID-19 and mental health. In this regard, this study reports three main results: (a) fear of COVID-19 significantly impacts on spirituality, (b) spirituality significantly impacts on mental health, and c) spirituality mediates the relationship between fear of COVID-19 and mental health.

#### Relationship Between Fear and Spirituality

Concerning the first hypothesis, this study revealed that fear and spirituality had a significant relationship on mental health. Such results indicate that even if people are afraid of COVID-19, the importance of faith will transcend this anxiety and it brings more positive vibration and compassion within themselves where it makes someone deal with their state of mental health. Such results support the findings of Polizzi et al. (2020), according to which many citizens were happy to uphold spirituality values in times of crisis. They agree that even such actions will offer them more positive values and contribute to better mental health.

#### Significant Relationship Between Spirituality and Mental Health

In accordance with the second hypothesis, the findings of the SMART PLS analysis conveyed that spirituality significantly effected on the mental health. This finding shows how important spirituality values are when looking into mental health among people, especially during the COVID-19 pandemic. In her writing, Rosmiran (2020) said that she spoke and encouraged her patients to always have faith and hope in overcoming their mental problems, especially during the COVID-19 pandemic. In this pandemic, many patients became worried about their futures, but hope and faith could make them more effectively transcend their state of mental health. This reveals how much faith ideals will improve more positive mental health, and this research shows how belief has more impact when it comes to mental health relative to anxiety.

#### Relationship Between Fear and Mental Health

Furthermore, the third hypothesis result found that fear of COVID-19 does not significantly effected on the mental health. People get worried quickly and are worried of diseases, especially during the COVID-19 pandemic. People are fearful easily because of the volume of social media information related to cases of people being hurt and dying every day. This condition is referred to as hypochondriatic anxiety disorder or health anxiety disorder. Fear and anxiety over well-being will trigger certain mental health problems, such as excessive stress and worries, and if ignored too, such doubts and suspicions can create many other mental health issues. But by spirituality, respondent’s fear of COVID-19 was overcome and their mental health reduced. It is the evidence based on this study, which clearly does not impact the skepticism of COVID-19 on mental health. COVID-19’s mistrust of faith impacts on mental health. It illustrates how spirituality values can help people deal with anxiety and mental health problems. Adults also need a lot of exposes to COVID-19 pandemic and how they can protect themselves (Rathakrishnan and Alfred, 2013). Adults who staying in rural area more are affected by their psycho well-being and mental health especially on COVID-19 pandemic (Rathakrishnan et al., 2019).

Finally, the fourth hypothesis found spirituality significantly mediated the relationship between fear of COVID-19 and mental health. This study affirms the importance of spirituality as a factor between fear and mental health. When spirituality values are high, people give less importance to emotion and become more focused on daily work. Even if people are experiencing social distancing and also having less social connectedness with their friends, family members, and colleagues, with spiritually, they can have faith and hope. This spirituality reduces their fear of disease. The result further shows that spirituality values could help people with fear to manage their mental health better. Spirituality makes them stronger in their relationship with fear and mental health. This result helps add to the overgrowing call for the mental health professional and counselor in hospital, school, and university. Fear of the disease can be reduced if spirituality well-being is imposed to increase positive mental health, especially throughout the COVID-19 pandemic. That means counselors and mental health professionals need to integrate spirituality well-being with fear and mental health. This spirituality values help adults to overcome fear, and it will increase positive mental health. Furthermore, this finding supports that counselor should consider spirituality values to reduce the number of people who have mental health and fear of COVID-19. An intervention to help adults who are having fear and mental health issue should focus on spirituality (Beckstein et al., 2021). This will help adults to increase their belief and positive values to control their fear and mental health issues (Liu et al., 2020, Rathakrishnan and George, 2021). Another essential point is that counselors and mental health professionals need to understand the role of spirituality in the life of the client (Seybold and Hill, 2001; Miller and Thoresen, 2003; Koenig, 2010). Counselors need to understand how to integrate their client’s spirituality values in their intervention and to help clients who have a fear of the COVID-19 and mental health issue. The process of intervention should focus on spirituality and values of positive well-being (Seligman, 2002).

#### Limitation

Justification in terms of location, time, or respondents’ characteristics also needs to be taken care. This study has the characteristics of a pilot study due to the limited sample size of adults. There should be more data collected.

#### Improvement

In future, research also can focus on more respondents and gives important of value of spirituality on the mental health and what type of invention using spirituality could ease the mental health among adults. Thus, it could be interesting in future occasions to assess the use of measures on other aspects of spirituality or religiosity (closeness to God, religious support.) and even introduce qualitative methodology.

## Conclusion

In conclusion, this study has identified the mediating role of spirituality in the relationship between fear of COVID-19 and mental health. The result demonstrated that there is a link between fear of COVID-19 and spirituality toward mental health. For future research, there is a need to conduct more research related to spirituality, fear, and mental health among people who are going through a COVID-19 pandemic. Fear of COVID-19 is an issue that needs to be explored to understand further about how it affects mental health among adults in Malaysia. Because people cannot be connected, it makes them more stressful and fearful of this disease. With more research and findings, there could be more counselors and mental health professionals who are culturally based and can tackle more issues related to mental health during times of a pandemic. This will help more counselors and mental professionals become well-equipped with more specific intervention methods for a pandemic.

## Data Availability Statement

The raw data supporting the conclusions of this article will be made available by the authors, without undue reservation.

## Ethics Statement

The studies involving human participants were reviewed and approved by Universiti Malaysia Sabah [JKEtika 3/20 (11)]. The patients/participants provided their written informed consent to participate in this study.

## Author Contributions

All authors listed have made a substantial, direct, and intellectual contribution to the work, and approved it for publication.

## Conflict of Interest

The authors declare that the research was conducted in the absence of any commercial or financial relationships that could be construed as a potential conflict of interest.

## Publisher’s Note

All claims expressed in this article are solely those of the authors and do not necessarily represent those of their affiliated organizations, or those of the publisher, the editors and the reviewers. Any product that may be evaluated in this article, or claim that may be made by its manufacturer, is not guaranteed or endorsed by the publisher.
